# Prevalence of elevated blood lead levels among pregnant women and sources of lead exposure in rural Bangladesh: A case control study

**DOI:** 10.1016/j.envres.2018.04.019

**Published:** 2018-10

**Authors:** Jenna E. Forsyth, M. Saiful Islam, Sarker Masud Parvez, Rubhana Raqib, M. Sajjadur Rahman, E. Marie Muehe, Scott Fendorf, Stephen P. Luby

**Affiliations:** a Emmett Interdisciplinary Program in Environment and Resources, Stanford University, Stanford, CA, USA; b Infectious Diseases Division, International Centre for Diarrhoeal Disease Research, Bangladesh, Dhaka, Bangladesh; c Earth System Science, Stanford University, Stanford, CA, USA; d Stanford Woods Institute for the Environment, Stanford University, Stanford, CA, USA

**Keywords:** Prenatal lead exposure, Case control study, Agrochemicals, Lead-soldered cans, Turmeric

## Abstract

Prenatal and early childhood lead exposures impair cognitive development. We aimed to evaluate the prevalence of elevated blood lead levels (BLLs) among pregnant women in rural Bangladesh and to identify sources of lead exposure. We analyzed the BLLs of 430 pregnant women randomly selected from rural communities in central Bangladesh. Fifty-seven cases were selected with the highest BLLs, ≥ 7 μg/dL, and 59 controls were selected with the lowest BLLs, < 2 μg/dL. An exposure questionnaire was administered and soil, rice, turmeric, water, traditional medicine, agrochemical, and can samples were analyzed for lead contamination. Of all 430 women, 132 (31%) had BLLs > 5 μg/dL. Most women with elevated BLLs were spatially clustered. Cases were 2.6 times more likely than controls to consume food from a can (95% CI 1.0–6.3, p = 0.04); 3.6 times more likely to use Basudin, a specific brand of pesticide (95% CI 1.6–7.9, p = 0.002); 3.6 times more likely to use Rifit, a specific brand of herbicide (95% CI 1.7–7.9, p = 0.001); 2.9 times more likely to report using any herbicides (95% CI 1.2–7.3, p = 0.02); and 3.3 times more likely to grind rice (95% CI 1.3–8.4, p = 0.01). Five out of 28 food storage cans were lead-soldered. However, there was minimal physical evidence of lead contamination from 382 agrochemical samples and 129 ground and unground rice samples. Among 17 turmeric samples, one contained excessive lead (265 μg/g) and chromium (49 μg/g). Overall, we found evidence of elevated BLLs and multiple possible sources of lead exposure in rural Bangladesh. Further research should explicate and develop interventions to interrupt these pathways.

## Highlights

• Prenatal blood lead levels and lead sources are assessed in rural Bangladesh.
• Elevated blood lead levels are found among pregnant women.
• Multiple possible sources of lead are identified; all related to food.
• Future lead prevention studies should investigate food storage and production.

## 1 Introduction

As a potent neurotoxin, lead (Pb) poses a serious threat to public health and human intellectual capital worldwide (Tong et al., 2000). After exposure via inhalation or ingestion, lead circulates in blood and is either excreted via urine or deposited in soft tissue or bone. The mean half-life of lead in blood is approximately 21–28 days, whereas lead accumulates in bones with a mean half-life of 5–19 years (Rabinowitz et al., 1976). During pregnancy, lead is mobilized from bones back into maternal blood and readily crosses the placenta into the blood of the developing fetus (Silbergeld, 1991, Röllin et al., 2009). Prenatal and early childhood lead exposures affect the developing central nervous system and produce irreversible cognitive damage that leads to adverse outcomes in adulthood (Bellinger, 2013).

Before the removal of lead in gasoline between the 1970–1990s, global blood lead levels (BLLs) were so high that the adverse effect of low levels of lead exposure was impossible to study (Bridbord and Hanson, 2009). For example, population mean BLLs dropped more than 75%, from 13–25 μg/dL to 2–3 μg/dL, in the US and South Korea within two decades after phasing out leaded gasoline (Pirkle et al., 1994, Oh et al., 2017). Subsequent multi-year cohort studies conducted in the US, Mexico, Australia, and Yugoslavia generated new evidence showing that lead exposure irreversibly decreases IQ, even at levels below 10 μg/dL (Ernhart et al., 1989, Baghurst et al., 1992, Bellinger et al., 1992, Dietrich et al., 1993, Wasserman et al., 1997, Schnaas et al., 2000, Canfield et al., 2003). A re-analysis of these data indicated that children with BLLs 2.4–10 μg/dL had IQ scores that were 3.9 points lower than children with BLLs < 2.4 μg/dL (Lanphear et al., 2005). In response to such evidence, the U.S. Centers for Disease Control and Prevention has continually lowered the threshold for elevated BLLs from 60 μg/dL in the 1960s to 5 μg/dL in 2015 (CDC, 2012, CDC, 2015). However, there is no known safe level of lead in the body.

Bangladesh phased out lead in gasoline in 1999, yet BLLs above 5 μg/dL persist across the country (Mitra et al., 2009, Mitra et al., 2012, Gleason et al., 2014). BLLs are comparatively higher in urban versus rural areas, though BLLs are higher than expected in non-industrial rural agrarian regions. In Dhaka, the capital city of Bangladesh, studies since 2000 have shown mean BLLs between 11.5 and 15 μg/dL among children under 16 years of age (Kaiser et al., 2001, Linderholm et al., 2011, Mitra et al., 2012). A 2007 study found 10 times higher BLLs in children living in industrial versus non-industrial neighborhoods in Dhaka (Mitra et al., 2009). Two studies from a rural agrarian region of Munshiganj district found high BLLs among more than 500 children under 4 years of age. In one study, 84% of children had BLLs ≥ 5 μg/dL and in another, the median BLL was 7.3 μg/dL (Gleason et al., 2014, Rodrigues et al., 2016). In Dinajpur, a different rural agrarian district, two studies reported similar BLLs, with means of 7.2 μg/dL (Mitra et al., 2009) and 7.3 μg/dL (Mitra et al., 2012) among a total of 380 children under 16 years of age. In this region, 25% of 16 parents had BLLs > 10 μg/dL (Mitra et al., 2009). In rural Narayanganj district, one study of 303 children 8–11 years of age reported a mean BLL of 11.5 μg/dL (Wasserman et al., 2011).

Multiple hypotheses have been explored in the literature for reasons why lead levels are high in rural Bangladesh but none have been conclusive. An analysis of lead exposure and BLLs among 919 children from both Dinajpur and Dhaka found that the mean BLL was 3.7 μg/dL higher for children whose families lived in close proximity to industries (p < 0.001) and 2.3 μg/dL higher for children whose families used certain traditional medicines compared to those who did not (p = 0.004) (Mitra et al., 2012). BLLs were also inversely correlated with body mass index (r = −0.23, p < 0.001) and hemoglobin levels (r = −0.10, p = 0.02). Low body mass index and low hemoglobin levels are indicators of poor nutrition. Nutrient deficient individuals, especially those lacking divalent metals like iron and calcium, absorb lead more readily, making them prone to lead poisoning (Goyer, 1995, Ahamed and Siddiqui, 2007). Other possible exposure sources included water source, metal taps, and melamine dinner plates, but were not significantly associated with BLLs after controlling for confounders.

Additional studies suggested agricultural and food-related exposure routes. Bergkvist et al. conducted a study of 408 pregnant women and 331 children in rural Chandpur district, concluding that rice may be an important source of lead due to contamination from agrochemicals (Bergkvist et al., 2010). The median rice lead concentration from 63 households was 0.013 μg/g. Based on an estimated consumption of 0.5 kg rice per day, median intake of lead from rice alone would be 6.5 μg/day, exceeding the established maximum daily intake limit set by the US Food and Drug Administration in 1993 (FDA, 2017). The 2013 Munshiganj study identified turmeric as a potential exposure route since 8 of 18 samples contained greater than 100 μg/g Pb (Gleason et al., 2014). Water, rice, and soil did not contain elevated lead concentrations in household samples and researchers did not attempt to draw statistical associations between BLLs and risk factors.

In an effort to evaluate the prevalence of elevated BLLs among pregnant women in rural Bangladesh and to identify the sources of lead exposure, we conducted a cross-sectional BLL assessment and a case control study.

## 2 Materials and methods

### 2.1 Study design and study population

**Fig. 1:**
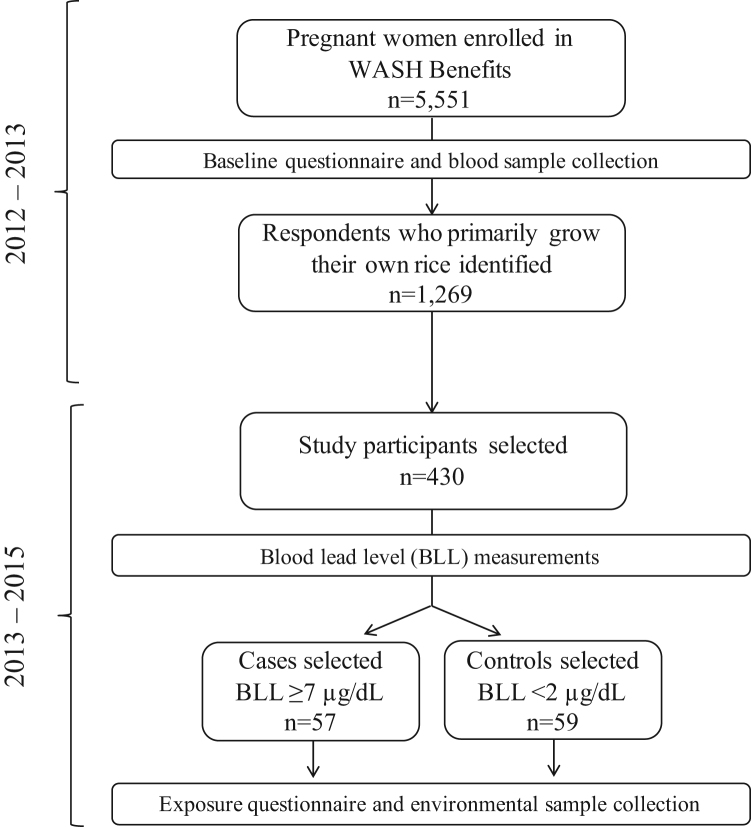
Overview of participant selection and research activities in Mymensingh, Tangail, and Kishoreganj districts between 2012 and 2015.

The case control study was nested within the WASH Benefits Bangladesh trial that began in 2012 and followed 5551 women from their first or second trimester of pregnancy through the first few years of their children's lives (Arnold et al., 2013). A sample size of 500 individuals was calculated to assess the prevalence of elevated BLLs among pregnant women enrolled in the WASH Benefits trial. For budgetary reasons, the sample was reduced to 430. A geographically stratified random sampling approach was used to select women from among all those who reported primarily consuming rice grown in their own field (Fig. 1, n = 1269). This inclusion criterion was chosen because our main hypothesis was that elevated BLLs in rural communities resulted from exposure to lead arsenate pesticide, and restricting to households who could report their own agrochemical use might provide a clearer signal of exposure. Study participants lived in Mymensingh, Tangail, and Kishoreganj districts. Based on the distribution of BLLs from the 430 women, 57 cases were selected who had the highest BLLs, ≥ 7 μg/dL, and 59 controls were selected who had the lowest BLLs, < 2 μg/dL. Our sample size of 116 provided 80% power to detect an odds ratio of 2.85 assuming 40% of controls are exposed. We based these power calculations on the hypothesis of a single predominant exposure pathway, that is lead arsenate pesticides.

### 2.2 Blood sampling and analysis

Between June 2012 to July 2013, before mothers were randomized to an arm of the WASH Benefits trial (Table 1, SI), research assistants collected 10 mL whole blood from all 5551 pregnant mothers using trace metal-free certified needles and tubes. Blood samples were diluted with reagent grade nitric acid and then BLLs were analyzed using Graphite Furnace Atomic Absorption Spectrophotometry (Shimadzu EX7, AA-6800) in the Nutritional Biochemistry Laboratory at icddr,b. We analyzed for spatial clusters of high and low BLLs by conducting an Optimized Hotspot Analysis using geographic information systems software.

**Table 1:** Demographic and select exposure characteristics from univariate analysis of 57 cases and 59 controls from Mymensingh, Tangail, and Kishoreganj districts, Bangladesh, 2014–2015.

Characteristic	Controls (%)	Cases (%)	OR (95% CI)
Age (years)^a^	24.0 ± 5.8	24.4 ± 4.8	1.01 (0.95–1.09)
Education (years)^a^	6.8 ± 3.6	6.5 ± 3.2	0.98 (0.88–1.10)
Gestational age (weeks)^a^	20.8 ± 5.6	20.3 ± 5.9	0.98 (0.92–1.05)
Body Mass Index (kg/m^2^)^a^, ^b^	20.8 ± 2.9	19.9 ± 3.3	1.10 (0.97–1.25)
Monthly income (taka)			
≤ 6000	15 (25.4)	13 (22.8)	1.00
6001–12,000	20 (33.9)	26 (45.6)	1.50 (0.58–3.86)
≥ 12,000	24 (40.7)	18 (31.6)	0.87 (0.33–2.26)
Owns a bicycle	26 (44.1)	22 (38.6)	0.80 (0.38–1.67)
Has electricity	41 (69.5)	35 (61.4)	0.70 (0.32–1.51)
Has tin roof	58 (98.3)	56 (98.2)	0.97 (0.06–15.82)
Number of rooms with tin walls			
0	12 (20.3)	10 (17.5)	1.00
1	43 (72.9)	35 (61.4)	0.98 (0.38–2.53)
> 1	4 (6.8)	12 (21.1)	3.60 (0.88–14.73)
Applied any paint in house in past year	13 (22.0)	7 (12.3)	0.50 (0.18–1.35)
Nearest road motor vehicles use (meters)			
< 50	18 (30.5)	20 (35.1)	1.00
51–200	20 (33.9)	18 (31.6)	0.81 (0.33–1.99)
> 200	21 (35.6)	19 (33.3)	0.81 (0.33–1.98)
Has a household member with risky occupation^c^	19 (32.2)	18 (31.6)	0.97 (0.44–2.12)
Wears kohl	4 (6.8)	5 (8.8)	1.32 (0.34–5.19)
Wears metal bracelet every day	51 (86.4)	54 (94.7)	2.82 (0.71–11.23)
Used traditional medicine in the past year	18 (36.7)	22 (43.1)	1.31 (0.59–2.92)
Purchases packaged turmeric	1 (1.7)	2 (3.5)	2.11 (0.19–23.92)
Consumes food from a metal can*	9 (15.3)	18 (31.6)	2.56 (1.04–6.33)
Closest brick kiln to agricultural field (km)			
0	4 (6.8)	3 (5.3)	1.00
1–2	30 (50.8)	27 (47.4)	1.20 (0.25–5.85)
3–10	25 (42.4)	27 (47.4)	1.44 (0.29–7.08)
Cultivates fish in own pond	32 (54.2)	36 (63.2)	1.45 (0.69–3.04)
Used pesticides last year	51 (86.4)	51 (89.5)	1.33 (0.43–4.12)
Used Basudin brand pesticide last year**	14 (23.7)	30 (52.6)	3.57 (1.61–7.90)
Used herbicides last year*	40 (67.8)	49 (86)	2.91 (1.15–7.34)
Used Rifit brand herbicide last year***	22 (37.3)	39 (68.4)	3.64 (1.69–7.86)
Grinds spices	54 (91.5)	53 (93.0)	1.23 (0.31–4.82)
Grinds rice*	34 (63.0)	45 (84.9)	3.31 (1.30–8.41)

a Mean ± SD reported for continuous variables.

b Body Mass Index from midline assessment (16 households missing).

c Risky occupation includes working with metal, paint, chemical waste, or batteries.

* =p-value < 0.05.

** =p-value < 0.01, and.

*** =p-value < 0.001.

### 2.3 Exposure questionnaire data collection and analysis

In November and December 2013, research assistants conducted an in-depth anthropological investigation involving semi-structured interviews with 10 heads of household and 10 women with BLLs > 10 μg/dL along with observations inside and outside each house to understand possible lead exposures and household characteristics in order to develop an exposure questionnaire. None of the houses were painted, inside or outside, which was typical of the study area. Between February and March 2014, research assistants administered the exposure questionnaire to all 116 case and control households. The questionnaire lasted approximately 45 min and covered up to 79 questions, depending on participants’ responses. Among these, up to 41 questions were related to general exposures from the literature and up to 38 were related to farming and agrochemicals.

Questionnaire data was analyzed using descriptive statistics and crude odds ratios (OR) for all exposures. For those exposures with a p-value < 0.05, we calculated the population attributable risk percent (PAR%) (Bruzzi et al., 1985).

### 2.4 Environmental sample collection and analysis

Research assistants collected physical samples over the course of 1.5 years to analyze for lead contamination. Soil, rice, turmeric, water, and traditional medicines were collected because of evidence from other studies. To collect soil, research assistants divided participants’ fields into a grid to randomly select sampling sites. Triplicate samples of soil were collected from the rooting depth of 25 cm (core soil) and the upper 2 mm (scraping soil). Uncooked rice was collected in resealable polyethylene bags. Research assistants used a random number generator to select a sub-sample of 10 case and 10 control households. From these 20 households, they collected turmeric and water from households’ wells in resealable polyethylene bags. Water was acidified upon analysis. Research assistants purchased at least one sample of every type of traditional medicine that participants reported using in the exposure questionnaire. This included 12 brands of herbal remedies.

Samples of cans, agrochemicals, and grinding mill rice were collected after the case control study identified elevated odds ratios for lead contamination associated with these exposures. Cans were collected from households who reported consuming food stored in cans. Research assistants purchased the seven most common types of agrochemicals identified in the exposure questionnaire from local stores in each of the eight sub-districts where study participants lived. Samples were collected every month between December through May when agrochemicals are used most frequently. Research assistants visited 15 rice grinding mills used by women with the highest BLLs who reported grinding rice at least three times per year and never consuming food stored in cans. Research assistants conducted semi-structured interviews with mill operators and collected samples of rice before husking (i.e., brown rice with the hull still on), before grinding (i.e., white rice with the hull removed), and after grinding (i.e., rice flour).

Soil samples from study participants’ fields were analyzed for lead concentrations via x-ray fluorescence (Spectro XEPOS HE, XLab Pro 5.1 software). Agrochemical samples were analyzed for lead concentrations via inductively coupled plasma mass spectrometry by California Laboratory Services (californialab.com). Samples of water, traditional medicine, rice, turmeric, and cans were analyzed for lead via inductively coupled plasma mass spectrometry (Thermo Scientific XSERIES 2 ICP-MS) at Stanford's Environmental Measurements Facility (em1.stanford.edu). Turmeric samples were also analyzed for chromium concentration. Samples of can seams that contained elevated lead concentrations above 100 μg/g were analyzed for the spatial distribution of lead within the can seams at the Stanford Synchrotron Radiation Lightsource (beamline 10-2); Pb and Fe were mapped at 0.5 mm step-sizes by the intensity of the fluorescent X-ray resulting from a primary beam of 13100 eV (calibrated to 13100 eV) with a pixel collection time of 20 ms. Fluorescence images of total Pb and Fe were processed with SMAK software (Webb, 2005). All analytical instruments are maintained and calibrated by dedicated staff. Standard laboratory procedures were followed including the analysis of blanks and reference materials, ensuring recovery up to 95% of the known Pb concentrations.

## 3 Results

**Fig. 2:**
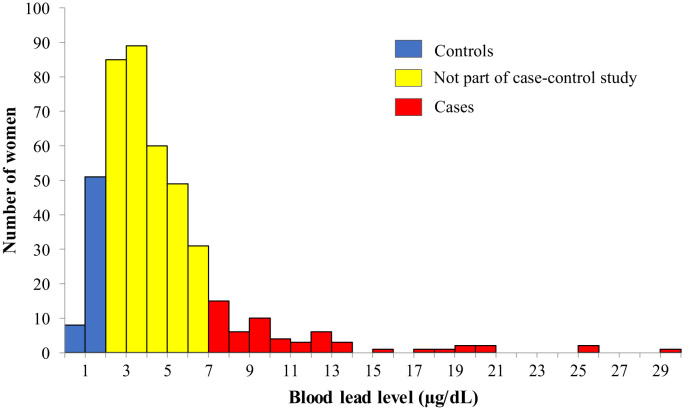
Distribution of blood lead levels among 430 randomly selected pregnant women from the WASH Benefits study in Mymensingh, Tangail, and Kishoreganj districts of rural Bangladesh who primarily consume rice from their own field, 2012–2013.

The distribution of BLLs was right-skewed with a mean of 4.7 (SD 3.6) μg/dL (Fig. 2). Of the 430 women, 31% had BLLs > 5 μg/dL and 6% of women had elevated levels > 10 μg/dL. The maximum BLL was 29.1 μg/dL, nearly 6 times greater than the CDC threshold for elevated BLL.

**Fig. 3:**
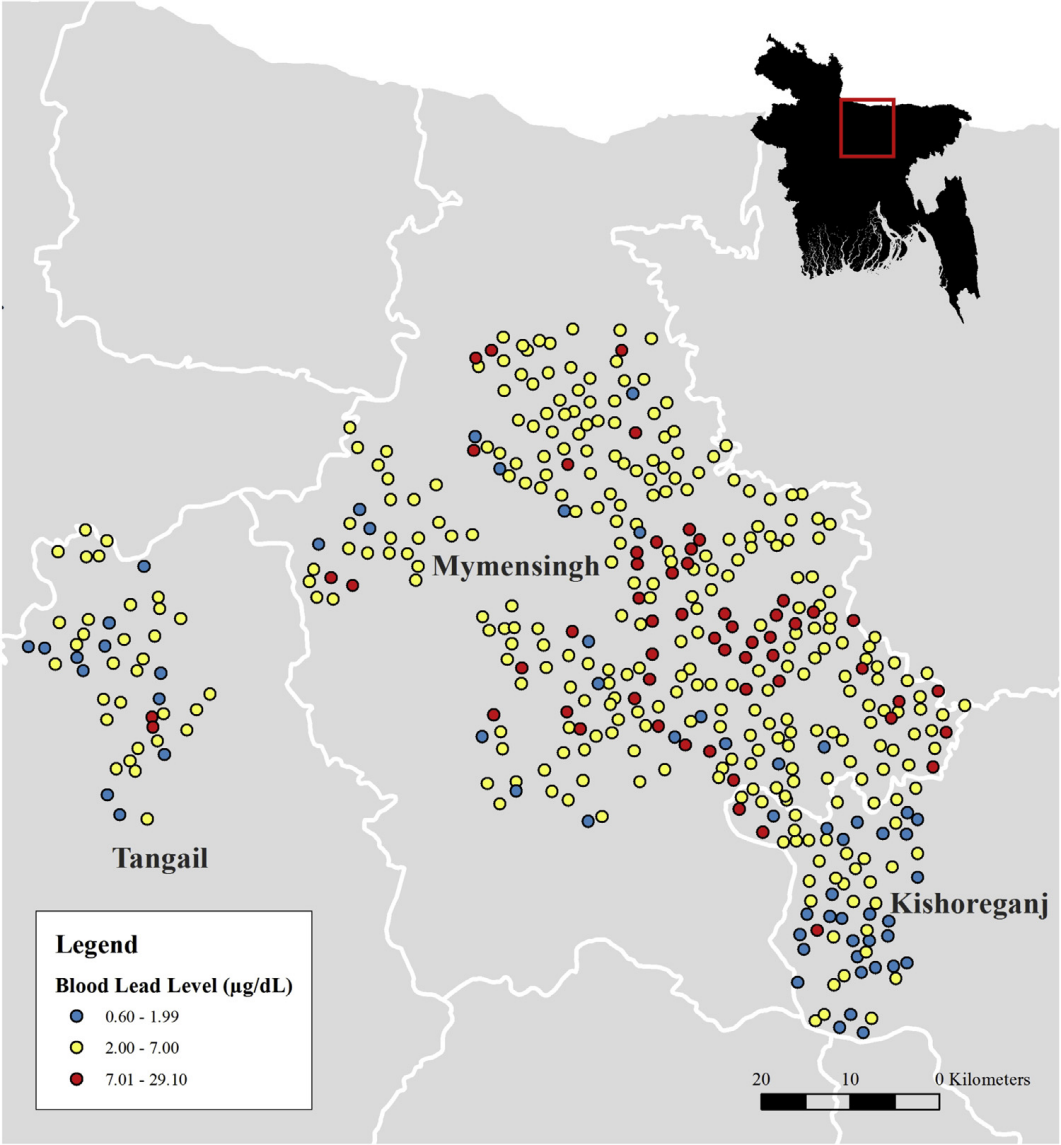
Spatial distribution based on defined categories of blood lead level among 430 randomly selected pregnant women from the WASH Benefits study in Mymensingh, Tangail, and Kishoreganj districts of rural Bangladesh who primarily consume rice from their own field, 2012–2013.

A total of 116 study participants were selected for enrollment in the case control study. Of these, 57 were cases, with BLLs ≥ 7 μg/dL, and 59 were controls with BLLs < 2 μg/dL (Fig. 2). Mean BLLs among controls were 1.4 (SD 0.4) μg/dL and 11.5 (SD 5.1) μg/dL among cases. Most cases (91%) lived in Mymensingh district, while the majority of controls (51%) lived in Kishoreganj district (Fig. 3). The Optimized Hotspot Analysis identified statistically significant non-random spatial clustering of women with high BLLs (hotspots) living in Mymensingh and women with low BLLs (coldspots) living in Kishoreganj (p-value = 0). Nonetheless, cases and controls did not exhibit differences in proximity to roads or indicators of wealth (Table 1; Table 2, Table 3, SI). Moreover, cases and controls were similar in gestational age, reported in weeks (OR 1.0, 95% CI 0.9–1.1); body mass index in kg/m^2^ (OR 1.1, 95% CI 1.0–1.3); and maternal age, with a mean of 24.0 (SD 5.8) years among controls and 24.3 (SD 4.8) among cases (OR 1.0, 95% CI 1.0–1.1). Less than one-third of cases and controls had any household members working with metal, paint, chemical waste, or battery recycling (OR 1.0, 95% CI 0.4–2.1). The use of paint was uncommon overall, and even less common among cases (12%) than controls (22%) (OR 0.5, 95% CI 0.2–1.4).

**Table 2:** Lead concentrations measured by ICP-MS of implicated exposure samples identified in Table 1 from 57 cases and 59 controls from Mymensingh, Tangail, and Kishoreganj districts, Bangladesh, 2014–2015.

Exposure pathway sample	N	[Pb] μg/g or mg/L	
		Median (IQR)	Min, max
Can seams^a^			
From cooking oil cans	6	2.3E+5 (1.3E+5–2.7E+5)	8.7, 3.9E+5
From other cans	22	4.5 (3.0−6.6)	<LOD, 92.2
Agrochemicals^b^			
Pesticides	192	<LOD (<LOD - 1.6)	<LOD, 8.3
Herbicides	140	<LOD (<LOD - 1.5)	<LOD, 6.3
Basudin	1	<LOD	<LOD
Rifit	49	<LOD	<LOD
Rice from grinding mill^a^			
Unhusked	6	0.3 (0.2–0.3)	<LOD, 0.9
Husked	9	<LOD	<LOD, 0.1
Ground	10	1.2E-2 (<LOD - 7.9E-2)	<LOD, 0.1

a Limit of detection (LOD): 0.001 μg/g Pb by ICP-MS.

b Limit of detection (LOD): 1.3 μg/g for solid samples and 0.5 mg/L for liquid samples by ICP-MS.

**Table 3:** Lead concentrations measured by ICP-MS or XRF of environmental samples from 57 cases and 59 controls from Mymensingh, Tangail, and Kishoreganj districts, Bangladesh, 2014–2015.

Sample	Both controls and cases			Controls			Cases		
	N	[Pb] μg/g or mg/L		N	[Pb] μg/g or mg/L		N	[Pb] μg/g or mg/L	
		Median (IQR)	Min, max		Median (IQR)	Min, max		Median (IQR)	Min, max
Soil^a^									
Scraping	112	29.4 (27.9–31.6)	21.8, 44.5	56	29.9 (28.3–31.6)	24.9, 42.4	56	28.9 (27.7–31.7)	21.8, 44.5
Core	112	29.2 (27.0–32.2)	20.1, 47.1	56	30.3 (27.8–31.8)	24.5, 43.1	56	28.2 (26.8–32.6)	20.1, 47.1
Uncooked rice^b^	102	<LOD	<LOD, 0.6	50	<LOD	<LOD, 4.6E-2	52	<LOD	<LOD, 0.6
Water^b^	20	<LOD	<LOD	11	<LOD	<LOD	9	<LOD	<LOD
Turmeric^b^	17	1.8 (1.1–4.4)	0.6, 264.9	10	1.8 (1.2–4.0)	0.6, 264.9	7	1.8 (1.1–4.9)	1.1, 6.5
Traditional medicine^b^	12	<LOD	<LOD, 0.2	–	–	–	–	–	–

a Limit of detection (LOD): 0.2 μg/g Pb by XRF.

b Limit of detection (LOD): 0.001 μg/g or mg/L Pb for solid or liquid samples by ICP-MS.

**Fig. 4:**
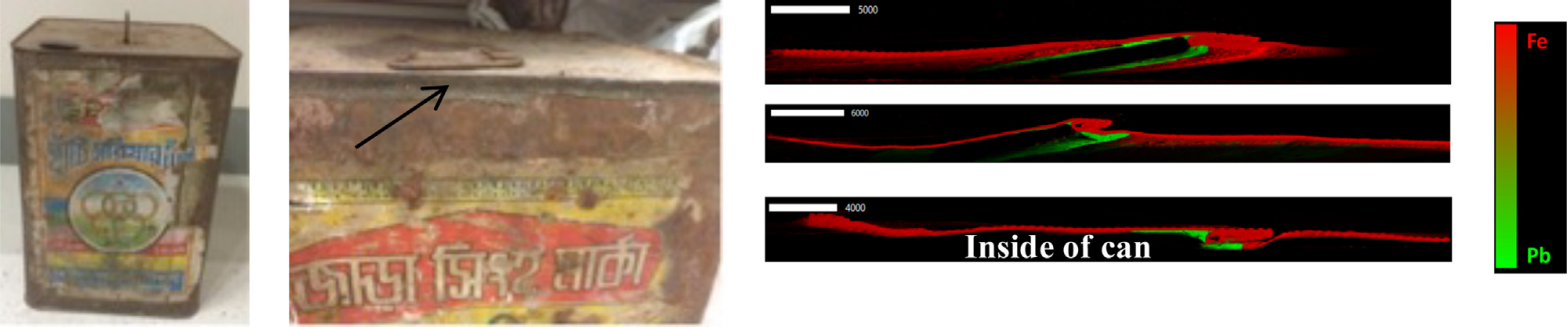
(Left) An image of one of the lead-soldered cans that previously held cooking oil; (middle) lead solder visible as designated by the arrow; (right) representative images of three can seams taken at Stanford's linear accelerator SLAC showing a cross-section of a can with lead solder (green) at seams facing the inside of the can and iron composing the can's walls (red). The elemental contribution of Pb and Fe in the cans was visualized through X-ray absorption at the respective elemental absorption edges of Pb L(III)-edge at 13035 eV and Fe K-edge 7112 eV. Normalization and dead time correction was done using the software Microanalysis Toolkit SMAK.

Compared with controls, cases were 2.6 times more likely to report consuming food from a can (95% CI 1.0–6.3, p = 0.04) (Table 1) with a population attributable risk of 7.5%. Twenty-eight cans were collected from 18 cases and 9 controls. Five of the 6 five-liter sized cans were recycled or repaired using lead solder at the seams (Fig. 4). The median concentration was 2.3E+ 5 μg/g or 23% Pb by weight (Table 2). Women stored various foods, mostly puffed rice, in these five-liter cans at the time of sample collection.

Cases were 3.6 times more likely than controls to use Basudin, a specific brand of pesticide (95% CI 1.6–7.9, p = 0.002); 3.6 times more likely to use Rifit, a specific brand of herbicide (95% CI 1.7–7.9, p = 0.001); and 2.9 times more likely to report using any herbicides (95% CI 1.2–7.3, p = 0.02) (Table 1). The population attributable risk ranged from 11.6% (Basudin use) and 18.4% (Rifit use) to 19.4% (any herbicide use). In total, 332 samples of agrochemicals (pesticides and herbicides) were collected, including 49 samples of Rifit but only one sample of Basudin was collected because it had been banned. Generally, agrochemicals were frequently re-packaged, suggesting that the contents of packages may not be reflective of the brand name. There was minimal evidence of lead contamination in the agrochemicals, with 70% of the samples having no detectable lead. None of the 49 samples of Rifit nor the sample of Basudin contained detectable lead. The maximum lead concentration for pesticides was 8.3 μg/g and 6.3 μg/g for herbicides (Table 2).

Cases were 3.3 times more likely to report grinding rice than controls (95% CI 1.3–8.4, p = 0.01) (Table 1) with a population attributable risk of 31.6%. Twenty-five samples of unhusked, husked, and ground rice were collected from local grinding mills associated with participants with the highest BLLs (Table 2). Half of the ground rice samples had a lead concentration above the level of detection, but the maximum lead concentration was only 0.1 μg/g. Grinding mill operators reported purchasing new mill stones instead of repairing cracked stones. According to qualitative assessments, households used different methods of grinding rice, but most of these methods involved wooden mallets or stones instead of metal.

**Fig. 5:**
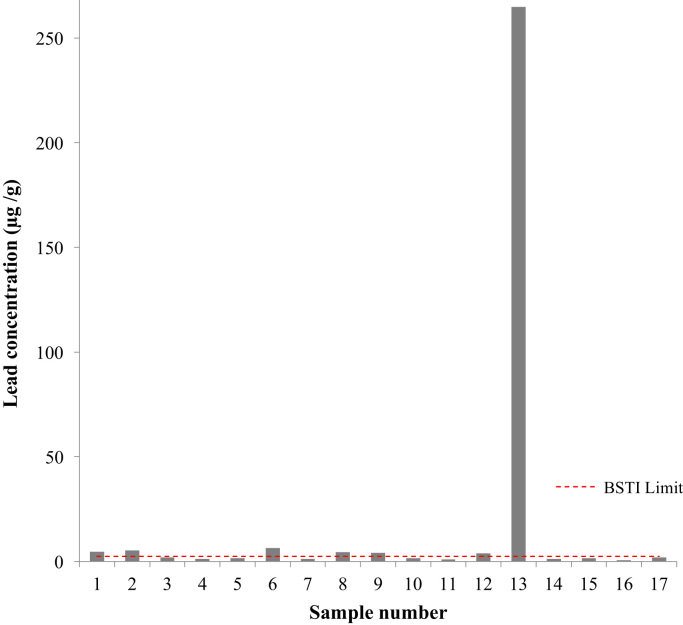
Lead concentration in 17 samples of turmeric from 20 randomly selected households. Seven of the 17 samples had a lead concentration greater than the Bangladesh Standards and Testing Institution's (BSTI) limit of 2.5 μg/g in turmeric.

Two cases and one control reported purchasing packaged branded turmeric (OR 2.1, 95% CI 0.2–23.9) (Table 1). Of the 17 analyzed turmeric powder samples, two were packaged turmeric and three individuals reported purchasing packaged turmeric (Table 1, Table 3). Seven of the 17 turmeric samples contained lead in excess of the Bangladesh Standards and Testing Institution's limit of 2.5 μg/g Pb in turmeric, though none were packaged. One of the unpackaged unbranded samples contained 265 μg/g lead (Pb) and 49 μg/g chromium (Cr), which is equivalent to a molar ratio of Pb:Cr of 1.3:1 (Fig. 5).

Cases were not more likely than controls to consume traditional medicines (OR 1.3, 95% CI 0.6–2.9) (Table 1). Only one of the 12 samples of traditional medicines, Cinkara brand multi-vitamin tonic, contained any detectable lead (0.2 μg/g) (Table 3).

There was minimal evidence of lead contamination among the other environmental samples from the participants’ own homes like soil, water, and rice. Median soil lead concentrations were low for both core and scraping soil samples (Table 3, 29 μg/g), and were not statistically different for cases and controls (Wilcoxon rank-sum test, p > 0.05). Lead concentrations of all soil samples were below the California standard of 80 μg/g in residential soils (OEHHA, 2018). None of the water samples and only 4 rice samples contained lead levels above the limit of detection of 0.001 mg/L or μg/g (Table 3). For reference, U.S. action levels for lead in water are set at 0.015 mg/L and although no rice-specific standards exist, 0.005 μg/g is the limit for lead in food intended for children (ATSDR, 2017).

## 4 Discussion

The BLL distribution among our study subjects living in three districts in central Bangladesh is consistent with studies from disparate smaller geographical areas. Considered together, this evidence suggests that pregnant women and children across rural Bangladesh are being exposed to unsafe levels of lead (Mitra et al. 2012; Gleason et al., 2014). The results from this case control study indicate that there are multiple sources of lead. Despite no known industrial or point sources of lead in the region, women with the highest BLLs lived near each other in Mymensingh district. We did not find any associations between BLL and proximity to roads or engagement in risky occupations (the majority of the population make their livelihood in agriculture). This suggests that there may be geographic heterogeneity to one or more of the multiple exposures we identified.

We found associations between elevated BLLs and the consumption of food from cans, use of agrochemicals, and rice grinding; we also found evidence of lead contamination in cans used for storing food and turmeric. We address each of these sources below by comparing the results from this study's exposure questionnaire and laboratory analyses with evidence from prior studies (Table 4).

**Table 4:** Summary of exposure questionnaire and laboratory evidence from this and other studies. A “+ ” indicates a positive association, a “-” indicates a negative association, and a “0” indicates no information.

	Questionnaire	Lab	Previous studies
Food storage cans	+	+	(Bolger et al., 1991, Bolger et al., 1996)
Agrochemicals	+	–	(Muhibbullah et al., 2005, Jayatilake et al., 2013)
Rice grinding	+	–	(Eisenberg et al., 1985, Koçak et al., 1989)
Turmeric	0	+	(Syed et al., 1987, Woolf and Woolf, 2005, Lin et al., 2010, Gleason et al., 2014)

Cans used for storing food were implicated based on evidence from the exposure questionnaire and physical evidence of lead solder. The cans were most commonly recycled or repaired with lead solder which could be a more common practice in some regions than others, leading to spatial clustering of BLLs. Historically, lead-soldered cans were common in high-income countries through the 1980s and 1990s. After the U.S. phased out lead-soldered cans in the 1980s, lead concentrations in infant formula dropped by 80%, representing a 20% decrease in overall child lead exposure (Bolger et al., 1991). Food stored in lead-soldered cans may absorb lead more or less readily depending on the chemical composition of the food, with acidic liquids absorbing the most (Bolger et al., 1996). In this study, women reported storing solid foods, particularly puffed rice, in the lead-soldered cans but lead could still be transferred from the cans through food to the mothers. One possibility is that since these cans are old and rusted, oxidized lead particles flake off with rust into puffed rice and are inadvertently consumed. Although the combined epidemiological and physical evidence for exposure to lead through cans is compelling, the population attributable risk fraction is only 7.5%, with 32% of cases and 15% of controls using cans to store food. Therefore, we conclude that other sources of lead also contributed to past and present human exposure.

Cases with elevated BLLs were more likely than controls to use Basudin, a diazinon-based organophosphate pesticide, and Rifit, a pretilachlor-based herbicide, and any herbicides on their rice fields. Throughout the first half of the 20th century, lead-based pesticides such as lead arsenate were the most popular pesticides in the world (Hood, 2006). Although officially banned in 1988 in the US, lead arsenate may have continued to be used in countries with limited regulatory capacity (Hood, 2006). Although we do not have physical evidence of lead contamination from any agrochemical in Bangladesh, we had limited opportunity to assess the lead concentration of Basudin since it was banned in 2013. One complicating factor is the informal market of re-packaging and re-labeling agrochemicals in Bangladesh. Nonetheless, if agrochemicals were a current pathway of exposure at the time of our assessment, we would have seen higher levels of lead contamination in soil and rice among cases who used agrochemicals more frequently than controls, which we did not find. The agricultural soil from cases and controls had similar levels of lead contamination and most of the rice grown in their fields had no detectable lead contamination.

Despite lack of lead in our agrochemical, soil, and rice samples, the exposure questionnaire suggests that agrochemicals, especially Basudin and Rifit, may have contributed to lead exposures in the past. Indeed, a study conducted over 10 years ago in rural Bangladesh found soil lead concentrations to be 2 times higher in agricultural plots compared to nearby non-agricultural plots and 1.5 times higher near the surface compared to a depth of 15–30 cm (Muhibbullah et al., 2005). However, soil lead concentrations were still below the California residential limit of 80 μg/g (OEHHA, 2018). A study in Sri Lanka found that one of 26 samples of pesticides and herbicides contained 931 μg/g Pb but the median value was 2 μg/g, suggesting that most samples had no detectable lead but a single type of agrochemical had excessive lead levels (Jayatilake et al., 2013). Given this heterogeneity found in Sri Lanka, even a single batch of lead-contaminated agrochemicals could contribute to spatial clustering of BLLs. Even though we did not confirm the presence of lead-tainted agrochemicals, the possibility remains that certain agrochemicals are occasionally contaminated with lead in Bangladesh and throughout South Asia due to variability in agrochemical sourcing or manufacturing processes.

Despite a strong association between the practice of rice grinding and BLL from the exposure questionnaire, we lack physical evidence to support this pathway as a source of ongoing exposure. Lead contamination from grinding wheat has been reported in Turkey and Israel, where lead solder was used to repair grinding stones (Eisenberg et al., 1985, Koçak et al., 1989). However, mill operators in the current study did not report repairing stones with lead solder. It is possible that grinding mills in this study repaired stones with lead solder in the past, but we did not find evidence of lead contamination in the rice samples after grinding. Even if one mill operator used lead solder for repair in the past, this could result in spatial clustering of high BLLs since only those consuming rice from that mill would be affected.

From our data, the prevalence of lead-contaminated turmeric is not clear, but other studies have suggested that this may be a problem throughout Bangladesh and the world (Woolf and Woolf, 2005, Lin et al., 2010). Because we only asked about packaged turmeric, we were underpowered to detect the association between turmeric and BLL through the exposure questionnaire. Nevertheless, 1 of 17 samples of turmeric powder contained chromium and more than 100 times the tolerable level of lead in spices. It is suspected that lead chromate, PbCrO_4_, is intentionally added to dried turmeric root and turmeric powder as a color enhancer (Gleason et al., 2014). The contaminated turmeric in our study also contained chromium with a molar ratio of lead to chromium of 1.3, close to the 1:1 ratio of pure lead chromate (PbCrO_4_). A study of turmeric from Bangladesh and Pakistan measured mean lead concentrations of 1.2 μg/g in raw fresh turmeric root (n = 3), 410 μg/g in dried polished turmeric root (n = 1), 290 μg/g in unbranded powdered turmeric (n = 3), and 30 μg/g in branded powdered turmeric (n = 3) (Syed et al., 1987). In 2013, the US FDA recalled Pran brand turmeric imported from Bangladesh due to elevated lead levels (FDA, 2013). However, the contaminated powdered turmeric from our study was unbranded.

This study highlights multiple exposures contributing to elevated BLLs and not a single exposure source. Consequently, our greatest limitation was that we were under-powered to identify uncommon or very common exposures. Although we were underpowered to detect an association between BLL and turmeric in the exposure questionnaire, this may be due to lack of power, because there is truly no association, or because of the way we asked the question in a way that did not allow for a nuanced analysis. We asked about consumption of branded packaged turmeric but did not distinguish based on the form of turmeric (raw fresh root, dried polished root, or powdered), which may be a better indicator of potential exposure.

The delay between time of exposure and time of assessment makes it difficult to implicate sources of lead. BLLs measured during pregnancy may be an indicator of cumulative exposure over a lifetime since lead is stored in the bones for decades and then released into the blood during pregnancy (Rabinowitz et al., 1976, Rabinowitz, 1991). Therefore, exposure to lead could have happened a decade prior to our exposure assessment and sample collection. Due to this temporal lag, we may have missed physical evidence of lead contamination from agrochemicals and ground rice. Testing samples for lead contamination is only informative when the current level of contamination is the same as the previous level. When products are banned or practices change, which may have occurred within the agrochemical and rice grinding pathways, physical testing of current exposures for lead contamination becomes uninformative.

## 5 Conclusions

Based on this and other studies, elevated BLLs appear to be a widespread problem in many districts across rural Bangladesh. There does not appear to be a single source of lead exposure in this context that can provide a simple focus for prevention, but rather several sources that require further investigation including lead-soldered cans, agrochemicals, rice grinding, and turmeric. Given the spatial clustering of women with elevated BLLs, prevention efforts could target geographic hotspots of lead exposure. Future case control studies should enroll a sufficiently large population to detect less common exposures and to assess the relative importance of each pathway. Although this study does not provide direct evidence, we suspect these exposures are not limited to Bangladesh. We recommend investigations in neighboring countries across South and Southeast Asia.

Moreover, we encourage further investigation into exposure-related practices, including how practices vary both temporally and spatially and how these exposures are driven by economic and social incentives. For the small percentage of the population that stores food in cans, solder from cans may be a source of persistent low doses of lead that accumulate over many years. Taking action to reduce exposure via this pathway will involve exploring the mechanism by which lead gets from the can to the food, why households store food in lead-soldered cans, where the lead solder comes from, what alternative lead-free solders exist, and what actions by government or other actors could limit the use of lead in repair and recycling activities. We suggest that researchers assess past and current agrochemical manufacturing practices to understand the possible addition of lead to agrochemicals. Similarly, more research to assess changes in grinding repair practices would highlight any potential for lead exposure from this pathway over time. We encourage that the issue of lead-adulterated turmeric be explored further since we were underpowered to assess this exposure and yet contaminated turmeric consumption could deliver high doses of lead, impacting not only South Asians but the rest of the world through the global supply chain. By systematically sampling and testing turmeric at different points in the supply chain, and exploring the incentives of the various actors, from farmers, to wholesalers, to retailers, to consumers, we could better identify at which point the lead adulteration may be occurring and where interventions are likely to be most productive.
