# Supplementary material for: Prevalence of elevated blood lead levels among pregnant women and sources of lead exposure in rural Bangladesh: A case control study
Source: Environ Res. 2018 Oct;166:1–9. doi: 10.1016/j.envres.2018.04.019 (PMC6143383; doi:10.1016/j.envres.2018.04.019)
Supplement: Supplementary file 1 — Supplementary material [file mmc1.docx]

**Supplemental Material**

**Prevalence of elevated blood lead levels among pregnant women and sources of lead exposure in rural Bangladesh: a case control study**

Jenna E. Forsyth,^1^ M. Saiful Islam,^2^ Sarker Masud Parvez,^2^ Rubhana Raqib,^2^ M. Sajjadur Rahman,^2^ E. Marie Muehe,^3^ Scott Fendorf,^3^ Stephen P. Luby^4^

^1^Emmett Interdisciplinary Program in Environment and Resources, Stanford University, Stanford, California, U.S.A.

^2^Infectious Diseases Division, International Centre for Diarrhoeal Disease Research, Bangladesh, Dhaka, Bangladesh

^3^Earth System Science, Stanford University, Stanford, California, U.S.A.

^4^Stanford Woods Institute for the Environment, Stanford University, Stanford, California, U.S.A.

**Corresponding author:** Jenna E. Forsyth, Emmett Interdisciplinary Program in Environment and Resources, 473 Via Ortega, Y2E2 Building, Suite 226, Stanford, CA 94305. Phone: 435-232-2955, email: jforsyth@stanford.edu

Table of Contents

[Table S1 2](#_Toc506466872)

[Table S2. 3](#_Toc506466873)

[Table S3 4](#_Toc506466874)

**Table S1.** Study participants according to enrollment in one of seven arms of the WASH Benefits study from Mymensingh, Tangail, and Kishoreganj districts, Bangladesh, 2013-2015.

|  | |  |  |  |
| --- | --- | --- | --- | --- |
| WASH Benefits Trial Arm | BLL Measured  N (%) | | Controls  N (%) | Cases  N (%) |
| Total | 430 (100) | | 59 (100) | 57 (100) |
| Control | 111 (25.8) | | 11 (18.6) | 15 (26.3) |
| Water | 53 (12.3) | | 11 (18.6) | 6 (10.5) |
| Sanitation | 50 (11.6) | | 8 (13.6) | 7 (12.3) |
| Handwashing | 57 (13.3) | | 5 (8.5) | 2 (3.5) |
| Water, Sanitation, and Handwashing (WSH) | 52 (12.1) | | 8 (13.6) | 12 (21.1) |
| Nutrition | 57 (13.3) | | 8 (13.6) | 9 (15.8) |
| Nutrition + WSH | 50 (11.6) | | 8 (13.6) | 6 (10.5) |
|  | | | | |

**Table S2.** Demographic characteristics from univariate analysis of 57 cases and 59 controls from Mymensingh, Tangail, and Kishoreganj districts, Bangladesh, 2014-2015.

| Characteristic | Controls (%) | Cases (%) | OR (95% CI) |
| --- | --- | --- | --- |
| Number of people in the compound |  |  |  |
| ≤ 9 | 20 (33.9) | 14 (24.6) | 1.00 |
| 10-14 | 25 (42.4) | 17 (29.8) | 0.97 (0.39 - 2.43) |
| 15-44* | 14 (23.7) | 26 (45.6) | 2.65 (1.03 - 6.81) |
| Number of years living in current location^a^ | 9.12 ± 15.5) | 8.26 ± 14.5 | 0.98 (0.93-1.04) |
| Monthly household income (taka per month) |  |  |  |
| ≤ 8,000 | 25 (42.4) | 23 (40.4) | 1.00 |
| 8,001-15,000 | 18 (30.5) | 21 (36.8) | 1.27 (0.54-2.98) |
| >15,000 | 16 (27.1) | 13 (22.8) | 0.88 (0.35-2.23) |
| Has electricity | 41 (69.5) | 35 (61.4) | 0.70 (0.32-1.51) |
| Has one or more wardrobes | 17 (28.8) | 13 (22.8) | 0.73 (0.32-1.69) |
| Has one or more tables | 53 (89.8) | 47 (82.5) | 0.53 (0.18-1.58) |
| Has one or more chairs | 51 (86.4) | 43 (75.4) | 0.48 (0.18-1.26) |
| Has one more bed (khat) | 46 (78) | 39 (68.4) | 0.61 (0.27-1.41) |
| Has one or more bed (chouki) | 43 (72.9) | 45 (78.9) | 1.40 (0.59-3.29) |
| Has radio | 2 (3.4) | 1 (1.8) | 0.51 (0.04-5.77) |
| Has television | 22 (37.3) | 18 (31.6) | 0.78 (0.36-1.67) |
| Has refrigerator | 5 (8.5) | 6 (10.5) | 1.27 (0.37-4.42) |
| Has bicycle | 26 (44.1) | 22 (38.6) | 0.80 (0.38-1.67) |
| Has motorbicycle | 8 (13.6) | 6 (10.5) | 0.75 (0.24-2.32) |
| Has sewing machine | 7 (11.9) | 4 (7) | 0.56 (0.15-2.03) |
| Has mobile | 50 (84.7) | 54 (94.7) | 3.24 (0.83-12.65) |
| ^a^Mean ± SD reported for continuous variables | | | |
| *=p-value <0.05, **=p-value <0.01, and ***=p-value <0.001 | | | |

**Table S3.** Exposure characteristics from univariate analysis of 57 cases and 59 controls with high and low blood lead levels (>7 and <2 μg/dL, respectively) from Mymensingh, Tangail, and Kishoreganj districts, Bangladesh, 2014-2015.

| Characteristic | Controls (%) | Cases (%) | OR (95% CI) |
| --- | --- | --- | --- |
| Wears metal earrings everyday | 48 (81.4) | 41 (71.9) | 0.53 (0.22-1.30) |
| Wears metal necklace everyday | 51 (86.4) | 54 (94.7) | 2.82 (0.71-11.23) |
| Wears metal nose pin everyday | 58 (98.3) | 55 (96.5) | 0.47 (0.042-5.38) |
| Wears metal amulet everyday | 24 (40.7) | 31 (54.4) | 1.74 (0.83-3.63) |
| Uses aluminum cooking pot | 59 (100) | 57 (100) | 1.00 |
| Uses plastic dishes | 59 (100) | 56 (98.2) | 1.00 |
| Uses metal dishes (steel, aluminum, and cast iron) | 59 (100) | 55 (96.5) | 1.00 |
| Uses clay dishes | 31 (52.5) | 35 (61.4) | 1.44 (0.69-3.01) |
| Uses painted clay dishes | 15 (25.4) | 24 (42.1) | 2.13 (0.97-4.69) |
| Consumes fish more than 4 days per week | 38 (64.4) | 28 (49.1) | 0.53 (0.25-1.12) |
| Grinds any crops and spices | 54 (91.5) | 53 (93) | 1.23 (0.31 - 4.82) |
| Grinds wheat | 6 (10.2) | 9 (15.8) | 1.64 (0.54 - 4.97) |
| Grinds coriander | 34 (57.6) | 19 (33.3) | 0.33 (0.15 - 0.72) |
| Grinds turmeric | 50 (84.7) | 43 (75.4) | 0.34 (0.10-1.18) |
| Grinds cumin | 2 (3.4) | 3 (5.3) | 1.56 (0.25 - 9.73) |
| Grinds red pepper | 53 (89.8) | 46 (80.7) | 0.12 (0.01 - 1.05) |
| Grows any spices* | 36 (61) | 21 (36.8) | 0.37 (0.18-0.79) |
| Grows crops other than rice | 58 (98.3) | 56 (98.2) | 1.00 |
| More than half of fruits come from own field | 19 (32.2) | 11 (19.3) | 0.5 (0.21-1.18) |
| More than half of vegetables come from own field | 12 (20.3) | 11 (19.3) | 0.94 (0.38-2.33) |
| More than half of rice comes from own field | 57 (96.6) | 52 (91.2) | 0.36 (0.067 - 1.96) |
| Number of fertilizer applications per year |  |  |  |
| ≤ 10 | 20 (33.9) | 23 (40.4) | 1.00 |
| 11-20 | 32 (54.2) | 26 (45.6) | 0.71 (0.32-1.56) |
| ≥ 21 | 7 (11.9) | 8 (14) | 0.99 (0.31-3.23) |
| Fertilizers used for rice |  |  |  |
| Triple super phosphate | 54 (91.5) | 47 (82.5) | 0.35 ( 0.10-1.18) |
| Diammonium phosphate | 15 (25.4) | 23 (40.4) | 1.94 (0.88-4.28) |
| Muriate of potash | 53 (89.8) | 50 (87.7) | 0.67 (0.2-2.26) |
| Gypsum | 25 (42.4) | 34 (59.6) | 1.95 (0.93-4.10) |
| Boron | 5 (8.5) | 2 (3.5) | 0.39 (0.07-2.07) |
| Vitamin | 23 (39) | 22 (38.6) | 0.96 (0.45-2.02) |
| Cow dung | 40 (67.8) | 42 (73.7) | 1.26 (0.56-2.83) |
| Fertilizers handled by study subject | 5 (8.5) | 12 (21.1) | 2.83 (0.93-8.63) |
| Fertilizers prepared inside the home | 17 (28.8) | 21 (36.8) | 1.44 (0.66-3.14) |
| Fertilizers prepared in the courtyard | 38 (64.4) | 35 (61.4) | .88 (0.41-1.87) |
| Number of pesticide applications last year |  |  |  |
| 0 | 8 (13.6) | 6 (10.5) | 1.00 |
| 1 | 30 (50.8) | 27 (47.4) | 1.27 (0.38-4.29) |
| >1 | 29 (49.2) | 30 (52.6) | 1.38 (0.43-4.47) |
| Pesticides used for rice |  |  |  |
| Diazinon | 6 (10.2) | 2 (3.5) | 0.32 (0.06-1.66) |
| Sunfuran | 4 (6.8) | 6 (10.5) | 1.62 (0.43-6.06) |
| Karate | 1 (1.7) | 2 (3.5) | 2.11 (0.19-23.92) |
| Furadan | 12 (20.3) | 2 (3.5) | 0.14 (0.03-0.67) |
| Virtako | 8 (13.6) | 5 (8.8) | 0.61 (0.19-2.00) |
| Pesticides handled by study subject | 0 (0) | 1 (1.8) | NA |
| Pesticides prepared inside the home | 6 (10.2) | 9 (15.8) | 1.65 (0.55-5.00) |
| Pesticides prepared in the courtyard | 22 (37.3) | 21 (36.8) | 0.98 (0.46-2.08) |
| Number of herbicide applications last year |  |  |  |
| 0 | 19 (32.2) | 8 (14) | 1.00 |
| 1 | 34 (57.6) | 42 (73.7) | 2.93 (1.14-7.52) |
| >1 | 6 (10.2) | 7 (12.3) | 2.77 (0.71-10.88) |
| Herbicides used for rice |  |  |  |
| Superhit | 4 (6.8) | 2 (3.5) | 0.50 (0.09-2.84) |
| Rifit | 22 (37.3) | 39 (68.4) | 3.64 (1.69-7.86) |
| Aimchlor 5G | 8 (13.6) | 4 (7) | 0.48 (0.14-1.70) |
| Herbicides handled by study subject | 0 (0) | 1 (1.8) | NA |
| Herbicides prepared inside the home | 1 (1.7) | 7 (12.3) | 8.12 (0.97-68.27) |
| Herbicides prepared in the courtyard | 12 (20.3) | 10 (17.5) | 0.83 (0.33-2.12) |
| Number of fungicide applications last year |  |  |  |
| 0 | 47 (79.7) | 49 (86) | 1.00 |
| 1 | 6 (10.2) | 3 (5.3) | 0.48 (0.11-2.03) |
| >1 | 6 (10.2) | 5 (8.8) | 0.80 (0.23-2.80) |
| Fungicides used for rice |  |  |  |
| Ridomol gold | 1 (1.7) | 1 (1.8) | 1.04 (0.06-16.96) |
| Jazz | 1 (1.7) | 1 (1.8) | 1.04 (0.06-16.96) |
| Fungicides handled by study subject | 0 (0) | 0 (0) | NA |
| Fungicides prepared inside the home | 0 (0) | 0 (0) | NA |
| Fungicides prepared in the courtyard | 1 (1.7) | 1 (1.8) | 1.04 (0.06-16.96) |
| *=p-value <0.05, **=p-value <0.01, and ***=p-value <0.001 |  |  |  |
